# Click-chemistry-derived oxime library reveals efficient reactivators of nerve agent-inhibited butyrylcholinesterase suitable for pseudo-catalytic bioscavenging

**DOI:** 10.1007/s00204-025-03985-6

**Published:** 2025-03-03

**Authors:** Tena Čadež, Nikolina Maček Hrvat, Goran Šinko, Jarosław Kalisiak, Zoran Radić, Valery V. Fokin, Karl Barry Sharpless, Palmer Taylor, Zrinka Kovarik

**Affiliations:** 1 Division of Toxicology, Institute for Medical Research and Occupational Health, Zagreb, Croatia; 2 Skaggs Institute for Chemical Biology, The Scripps Research Institute, La Jolla, CA USA; 3 Skaggs School of Pharmacy and Pharmaceutical Sciences, University of California at San Diego, La Jolla, CA USA; 4 The Bridge@USC and Loker Hydrocarbon Research Institute, University of Southern California, Los Angeles, CA USA; 5 Faculty of Science, University of Zagreb, Zagreb, Croatia

**Keywords:** Antidotes, Organophosphorus compounds, 2-PAM, Obidoxime, Acetylcholinesterase

## Abstract

A library of 100 click-chemistry-derived oximes was evaluated as reactivators of butyrylcholinesterase (BChE) inhibited by the nerve agents (NAs) sarin, cyclosarin, VX, and tabun. While reactivation efficiency was highly dependent on the structure of both the NA and the oxime, for each NA-BChE conjugate, we identified reactivators more effective than currently approved oximes for NA poisoning. Detailed kinetic analysis indicated that this enhancement results from both improved molecular recognition—specifically, enhanced binding affinity of the phosphylated conjugates for the oximes—and increased maximal reactivation rates. Molecular modeling of oximes in a near-attack conformation within inhibited BChE revealed critical interactions for productive reactivation. Among all tested oximes, 5B [1-hexyl-2-((hydroxyimino)methyl)pyridinium chloride] emerged as a particularly efficient reactivator for BChE phosphorylated with cyclosarin, with the highest observed overall reactivation rate of 34,120 M^−1^ min^−1^, which is 525-fold and 44-fold higher than the reference oximes 2-PAM and HI-6, respectively. In general, three mono-pyridinium mono-oximes demonstrated more efficient recovery of BChE activity than bis-pyridinium triazole-annulated click-chemistry bis-oximes, which were previously identified as potent reactivators for inhibited acetylcholinesterase (AChE). Ex vivo assessment of reactivation potency demonstrated that the combined addition of BChE with one efficient reactivator for BChE and another for AChE achieved > 90% reactivation of cyclosarin-inhibited cholinesterases in whole blood (WB), demonstrating near-complete degradation of a 100-fold excess of cyclosarin within 6 min. These results confirm that oxime-assisted catalysis is feasible for NA bioscavenging in blood and underscore BChE’s potential as a target for developing therapies against NA poisoning.

**Supplementary Information:**

The online version contains supplementary material available at 10.1007/s00204-025-03985-6.

## Introduction

Oxime reactivators essential for the nucleophilic displacement of the phosphorus moiety from organophosphorus compounds (OP)-inhibited cholinesterase active site present an eminent segment in current medical countermeasures for exposure to OPs. OPs as pesticides or nerve agents (NA) are potent inhibitors that progressively deactivate acetylcholinesterase (AChE; EC 3.1.1.7) by covalently binding to the enzyme’s catalytic serine leading to the accumulation of neurotransmitter acetylcholine (ACh) in cholinergic and neuromuscular synapses. Constant depolarizations of postsynaptic neurons can induce a potentially fatal syndrome known as a cholinergic crisis, characterized by symptoms of respiratory depression, cardiopulmonary arrest, and convulsions (Vale et al. 2016; Timperley et al. 2019a; Kovarik et al. 2024; Voros et al. 2024). Oxime-based antidotes, such as pralidoxime (2-PAM), obidoxime, asoxime (HI-6), and trimedoxime (TMB-4), have been approved for treatment of acute exposure to OPs (Maček Hrvat and Kovarik 2020). The primary limitation of these reactivators is their narrow effectiveness against a broad range of OPs, poor ability to penetrate the blood–brain barrier (BBB) at sufficient concentrations, and ineffectiveness in reactivating butyrylcholinesterase (BChE, EC 3.1.1.8) (Zorbaz and Kovarik 2020; Prchalova et al. 2023). The inadequacy of current therapy in the case of OP poisoning has led to the exploration of alternative countermeasures based on BChE which can bind OP inhibitors, the same as its related enzyme AChE, thereby reducing OP concentration and providing protection to the cholinergic system from inhibitory effects (Reed et al. 2017; Timperley et al. 2019b). Therefore, BChE serves as an endogenous bioscavenger as it gets irreversibly inhibited by binding one molecule of OP (Allard et al. 2022). Indeed, several studies have demonstrated that the administration of BChE derived from human plasma enhances survival following exposure to multiple lethal doses of tabun, VX, sarin, or soman, and alleviates toxic symptoms in animals without the need for additional therapy (Myhrer and Aas 2016). However, attaining similar outcomes in human subjects requires a substantial amount of BChE to eliminate OPs from circulation (Steindl et al. 2021). Reduction of the required BChE concentration can be achieved by forming a pseudo-catalytic complex between BChE and its effective oxime reactivator (Kovarik et al. 2010). This oxime-assisted (pseudo-catalytic) bioscavenger could ensure the degradation of a substantial quantity of OP molecules through repeated cycles of inhibition and reactivation (Kovarik et al. 2007a, 2015, 2019a; Masson and Nachon 2017; Kovarik and Maček Hrvat 2020; Radić et al. 2013). To successfully develop such a system, a highly effective oxime is required, capable of rapidly dephosphorylating BChE at a high rate and at low concentration.

In this context, oxime structure plays a crucial role in the reactivation efficacy of inhibited cholinesterases. Standard oximes have not demonstrated efficacy in reactivating inhibited BChE as they have for inhibited AChE (Kohoutova et al. 2024). To address this, new reactivators for NA-BChE conjugates have been designed and synthesized including oximes based on the BChE inhibitor edrophonium (Radić et al. 2013), triptoline-based oximes and furan-based oximes (Renou et al. 2014; Gorecki et al. 2024), imidazolium oximes (Gorecki et al. 2024; Sit et al. 2014; Katalinić et al. 2016), quinuclidine oximes (Zandona et al. 2020), olesoxime (Kolić et al. 2024), pyridinium (Lucić Vrdoljak et al. 2006) as well as halogenated pyridinium oximes (Zorbaz et al. 2019; Amitai et al. 2021), symmetrical oximes with isoquinine units (Lee et al. 2021), and hydroxyiminoacetamid oximes (Gorecki et al. 2024; Maraković et al. 2020).

Also, a rapid synthesis of oximes achieved by click chemistry using Cu(I)-catalyzed azide-alkyne cycloaddition (Rostovtsev et al. 2002), a reaction between an acetylenic and azide building blocks to form triazoles with an appropriate oxime functionality enabled the generation of a library of various oxime compounds (Cochran et al. 2011). Among them, triazole compounds showed to be high-affinity AChE inhibitors with an exceptional binding affinity in the micro- to femtomolar range (Manetsch et al. 2004; Krasiński et al. 2005; Maček Hrvat et al. 2020).

**Fig. 1:**
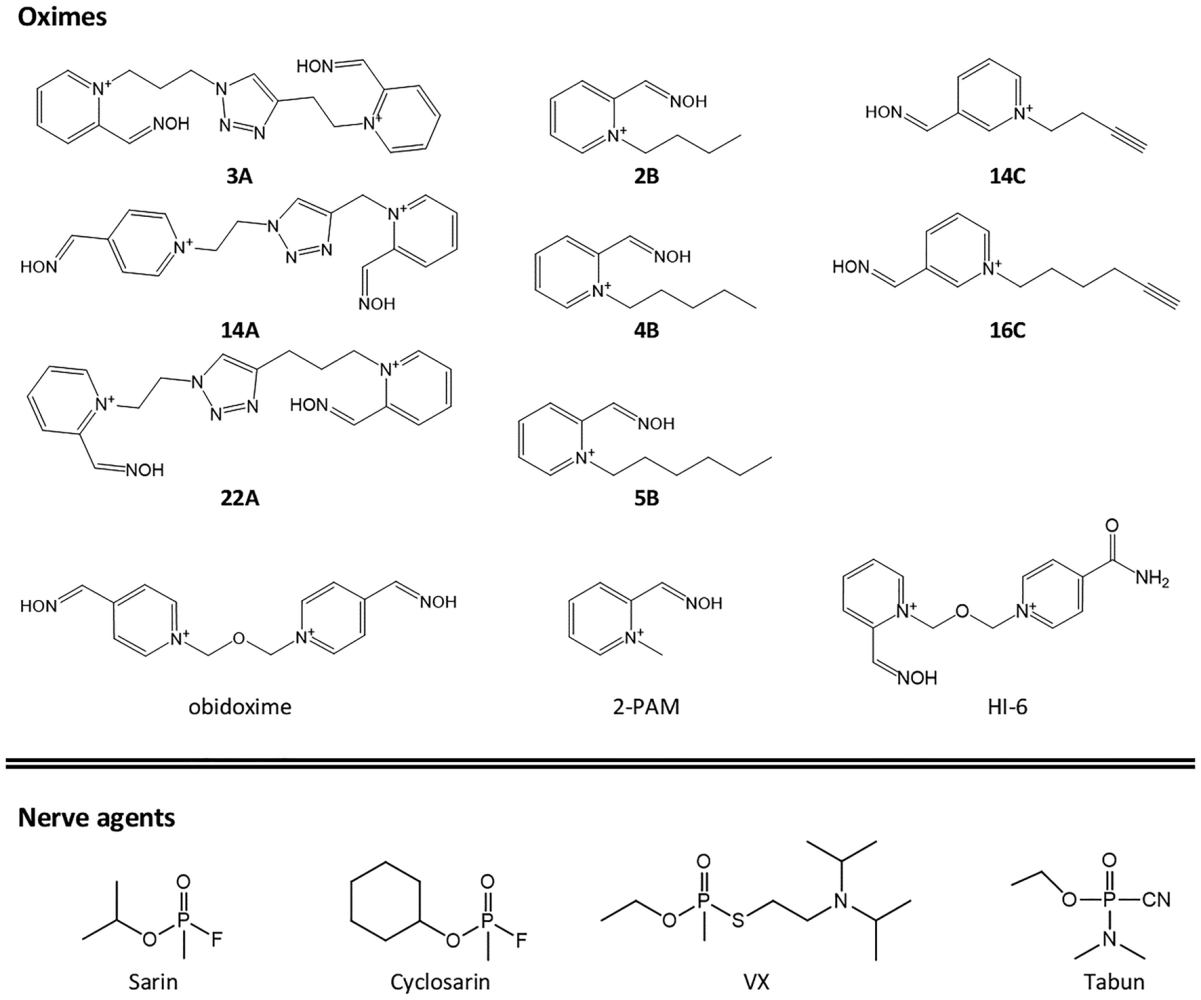
Structures of selected click-chemistry-derived oximes, standard oximes, and nerve agents used in this study

In this study, we employed a click-chemistry-derived oxime library comprised of triazole-annulated oximes and their building blocks, previously tested as AChE reactivators (Kovarik et al. 2019b). Oximes were screened for oxime-dependent reactivation of sarin-, cyclosarin-, tabun-, and VX-inhibited human BChE. A comprehensive in vitro analysis of enzyme kinetics, enabled detection of more efficient reactivators of phosphylated BChE than obidoxime, HI-6, or 2-PAM (Fig. 1). Several novel oximes were particularly efficient in the reactivation of cyclosarin–BChE conjugate. Reactivation in ex vivo conditions proved the feasibility of OP bioscavenging in the blood by an efficient reactivator of BChE. We also estimated reversible inhibition constants of complexes with AChE and BChE for selected oximes constants reflecting their binding affinities. Furthermore, computational docking was used for modeling a near-attack conformation of oxime in the cyclosarin-inhibited BChE revealing different oxime positioning in the active sites of inhibited and native BChEs.

## Materials and methods

### Chemicals and enzymes

Most of 111 compounds in the oxime library were synthesized using the click-chemistry method, a copper-catalyzed cycloaddition between azide and alkyne structural units with the formation of a triazole ring, as previously described (Cochran et al. 2011; Kovarik et al. 2019b) (all structures are presented in Table 1). The original oxime solutions were prepared as 100 mM in distilled water and stored at 4 °C, except 1A, 3A, 7A, 8A, 9A, 16A, 17A, 22A, 25A, 28A, 32A, 38A, 40A, 44A, 50A, 56A, 4C, 12C, 17C, 3D, 5D, which were dissolved in dimethyl sulfoxide (DMSO; Kemika, Zagreb, Croatia). Further dilutions were prepared in distilled water immediately before the experiment. Nerve agents, cyclosarin, sarin, VX, and tabun were purchased from NC Laboratory, Spiez, Switzerland. NA stock solutions were prepared in isopropyl alcohol (Sigma-Aldrich, St. Louis, MO, USA), while further dilutions were made in distilled water immediately before use. Acetylthiocholine iodide (ATCh), 5,5′-dithiobis(2-nitrobenzoic acid) (DTNB), and bovine serum albumin (BSA) were purchased from Sigma-Aldrich (St. Louis, MO, USA).

**Table 1:**
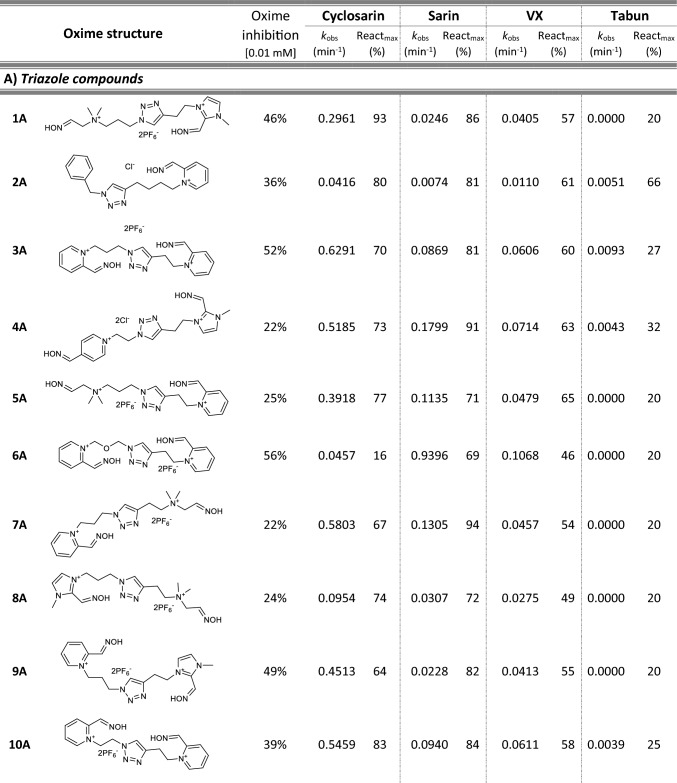

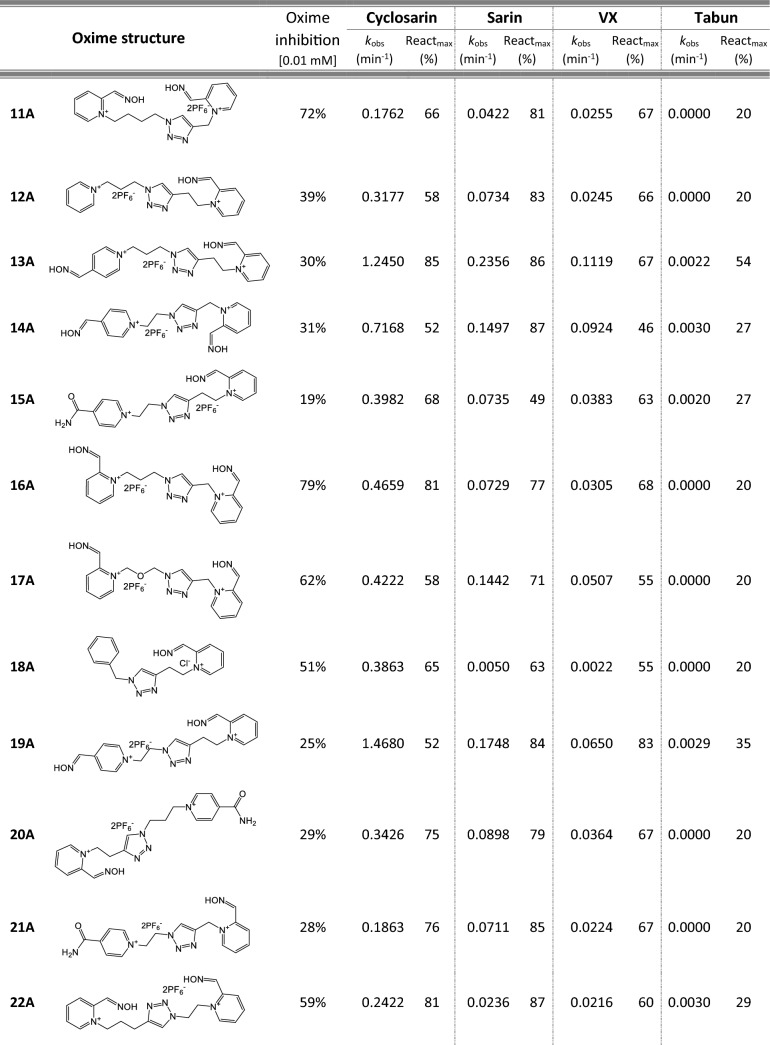

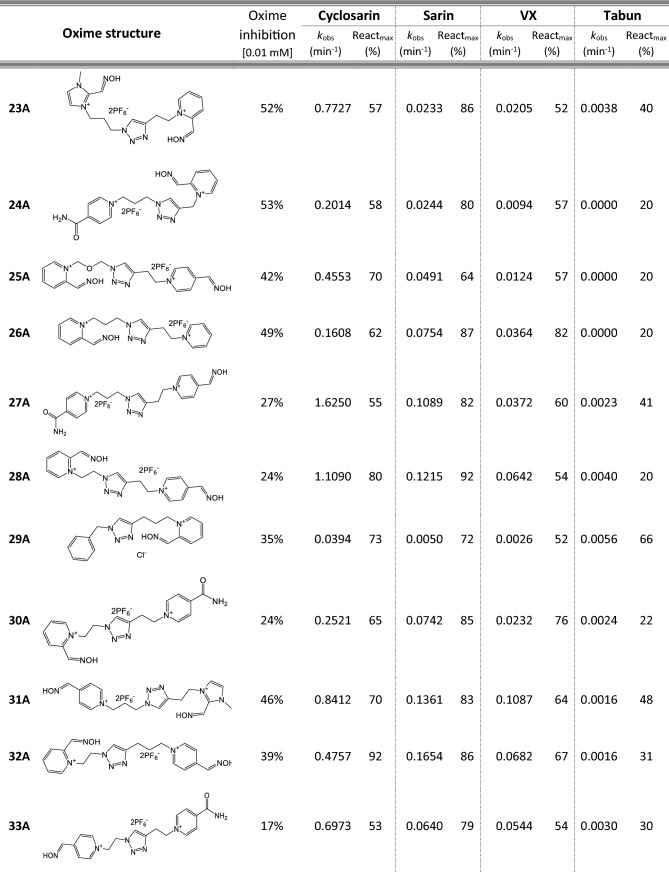

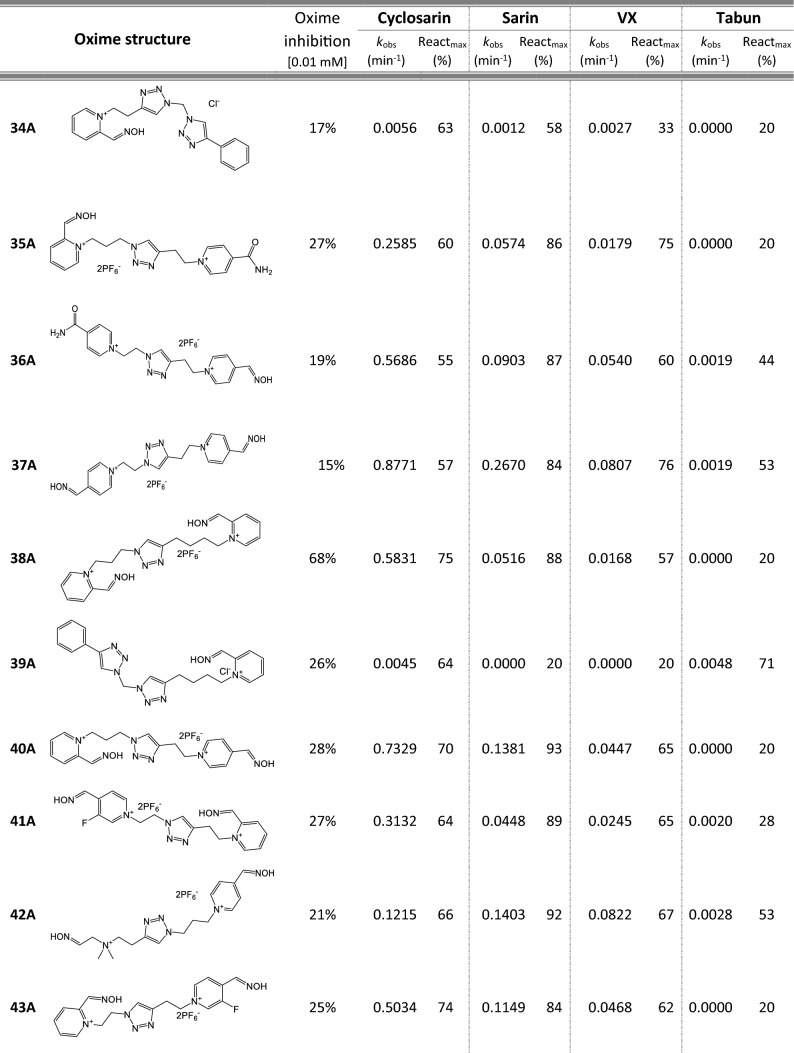

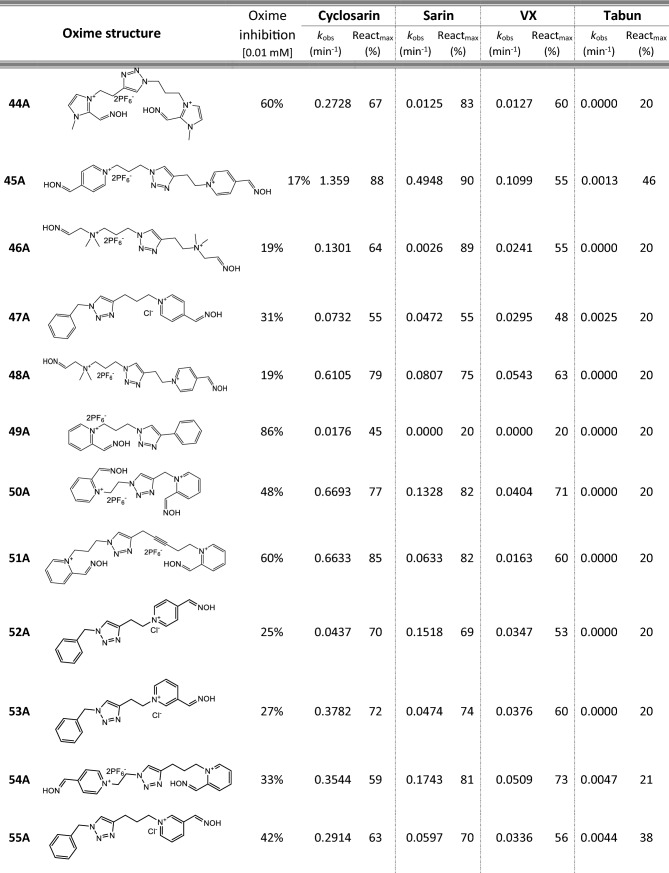

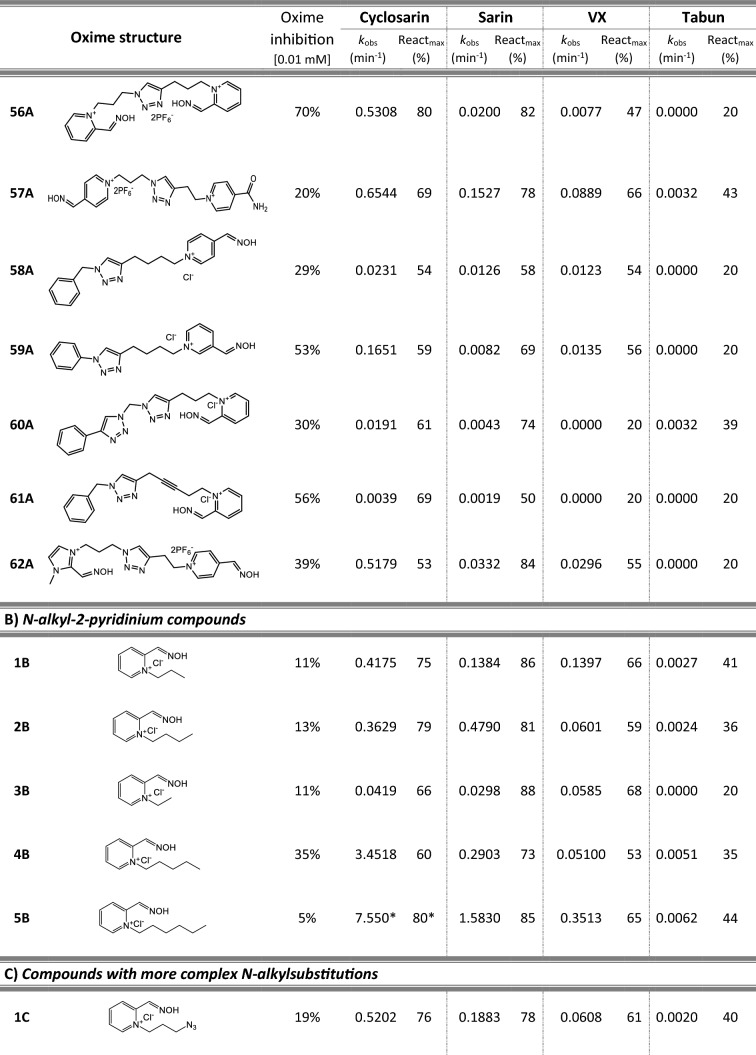

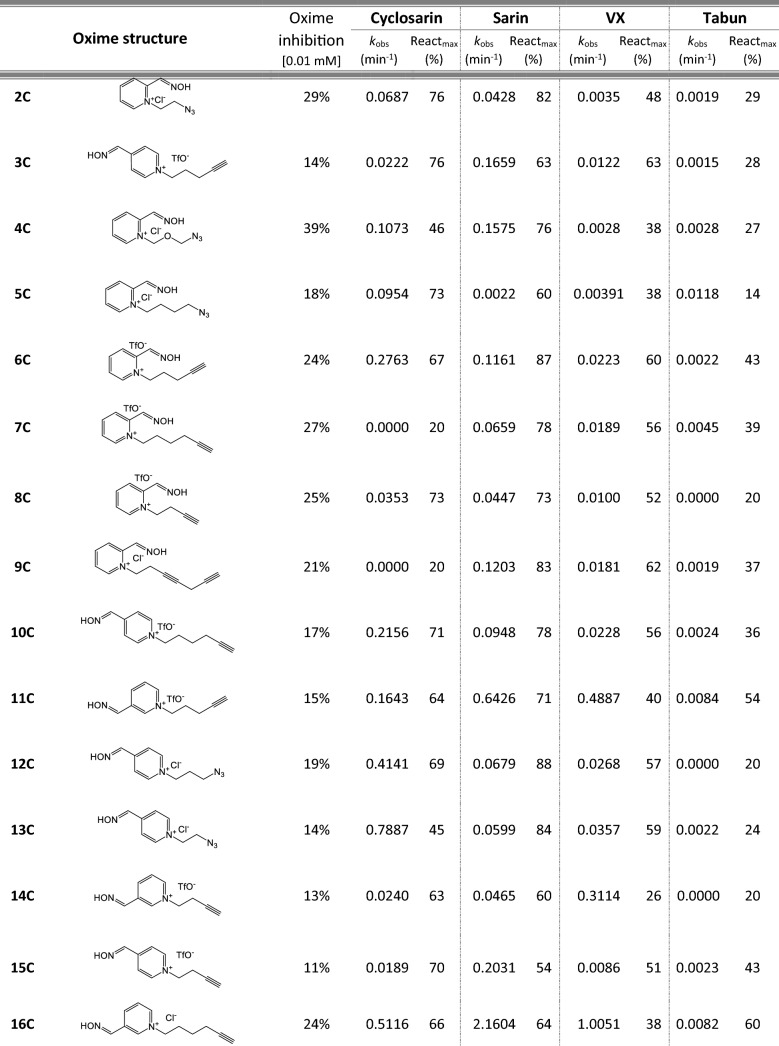

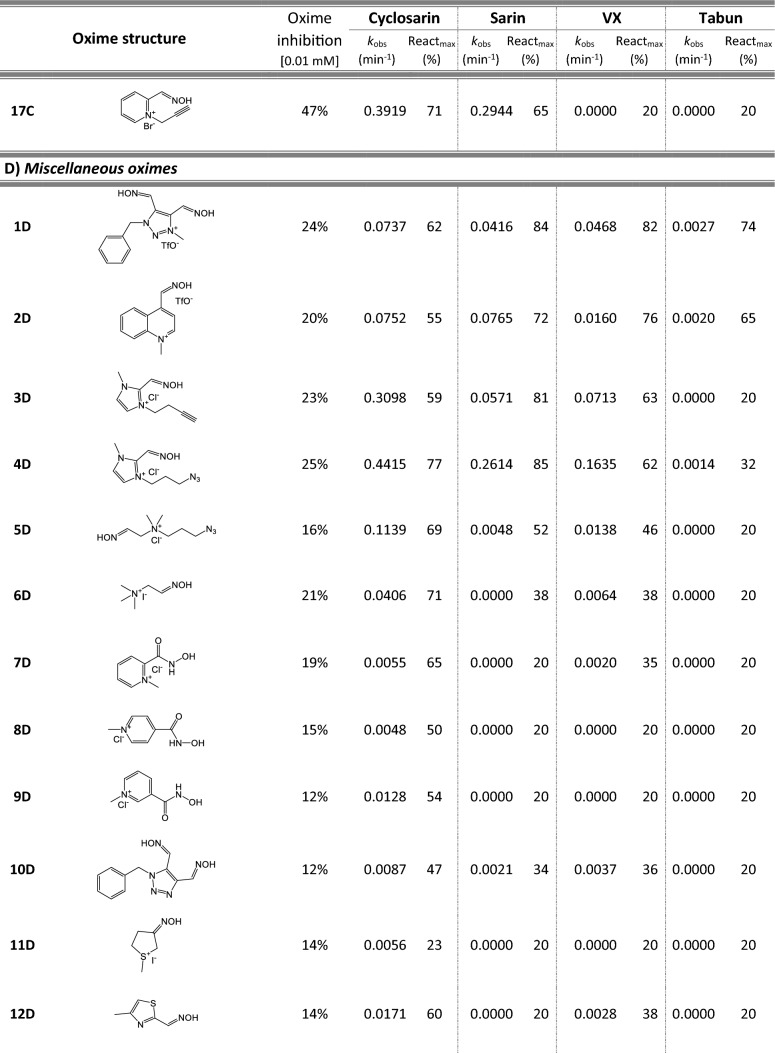

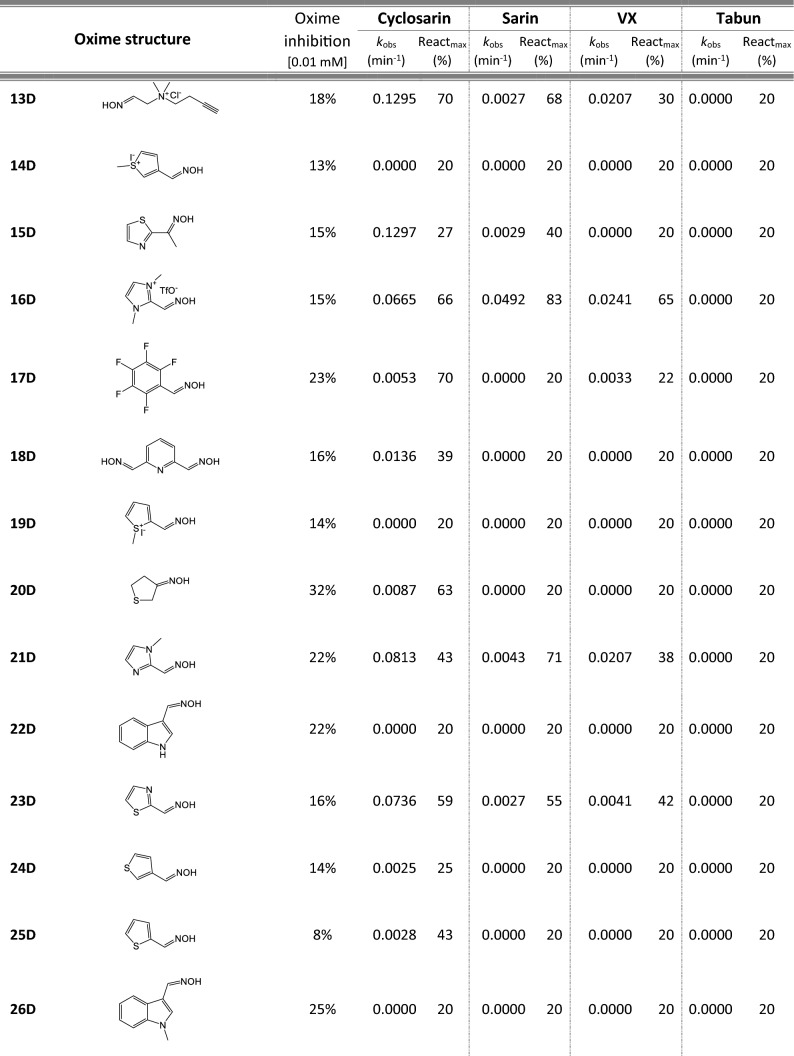

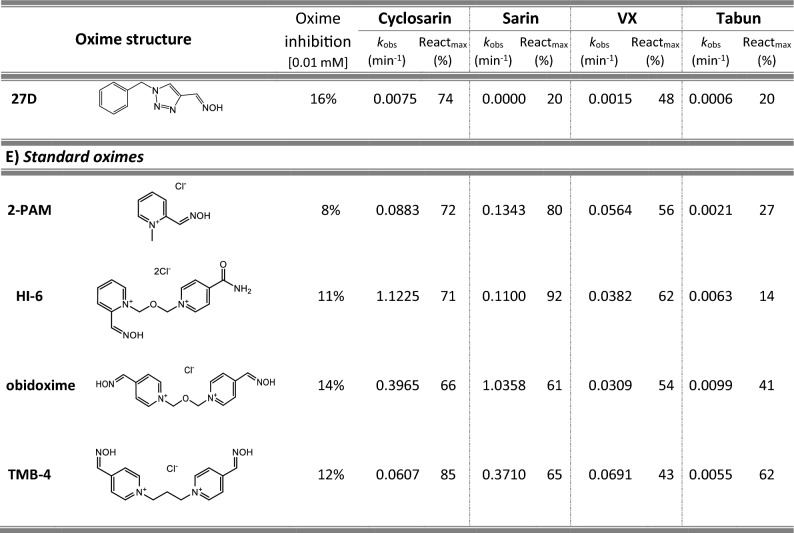
Reactivation screening of the NA-inhibited BChE by (A) 62 triazole compounds, (B) 5 *N*-alkyl-2 pyridinium compounds, (C) 17 compounds with more complex *N*-alkyl substitutions, (D) 27 miscellaneous oximes, and (E) four standard oximes

The reactivation rate, *k*_obs_ and maximal percentage of reactivation, React_max_, were obtained at the 1 mM oxime concentration (except oxime 5B for cyclosarin were oxime concentration was 0.1 mM). Reactivation was monitored up to 24 h. Inhibition of native BChE with 0.01 mM concentration of oxime was also displayed

*Concentration of oxime 5B was 0.1 mM

For in vitro kinetic experiments, recombinant human AChE and BChE purified from human plasma were used. The recombinant human AChE wild type was prepared as described earlier (Cochran et al. 2011), while purified human plasma BChE was a gift from Dr. Florian Nachon, *Département de Toxicologie et Risques Chimiques, Institut de Recherche Biomédicale des Armées*, Brétigny-sur-Orge, France. Relevant concentrations of AChE and BChE were prepared in a 0.1 M phosphate buffer pH 7.4 and supplemented with 1% BSA in the case of AChE.

For ex vivo experiments, human whole blood (WB) was collected from a healthy female donor at the Institute for Medical Research and Occupational Health, Zagreb, Croatia following approval by the Ethics Committee of the Institute.

### Reactivation of inhibited cholinesterases

For reactivation experiments, AChE or BChE were inhibited with a tenfold excess of NA to completely inhibit the enzymes in 1 h. This mixture was filtered using a Sephadex G-50 spin column (Roche Diagnostic GmbH, Mannheim, Germany) to remove the excess of unconjugated NA, and then diluted in 0.1 M sodium phosphate buffer, pH 7.4 (in case of AChE also containing 0.01% BSA). Afterward, the aliquots were incubated with oxime to initiate the reactivation reaction at 25 °C. At specified time intervals, aliquots were diluted (100 X) in phosphate buffer containing DTNB, and upon the addition of ATCh, residual enzyme activity was measured using the Ellman method (Ellman et al. 1961). The same procedure and oxime were applied to the uninhibited reaction mixture, where an appropriate volume of distilled water was added instead of the NA and was used as a control. The final concentrations of ATCh and DTNB were 1.0 and 0.3 mM, respectively. Control and reactivation reactions were corrected for oximolysis (Šinko et al. 2007).

An oxime concentration of 1 mM was used in screening to determine the maximal percentage of reactivation (React_max_) and the first-order reactivation rate constant at a given concentration (*k*_obs_) when React_max_ was greater than 20%. For detailed kinetic experiments, a wide range of oxime concentrations was employed to determine: the maximum reactivation rate constant (*k*_2_), the dissociation constant (*K*_OX_), and the overall reactivation rate constant (*k*_r_), as previously described (Kovarik et al. 2004). Reactivation experiments were performed at 25 °C, using the Ellman spectrophotometric method at 412 nm on a CARY 300 spectrophotometer (Varian Inc., Mulgrave, Australia), and the results were analyzed with Prism 9 software (GraphPad by Dotmatics, Boston, MA, USA).

### Reversible inhibition of cholinesterases

Reversible inhibition of cholinesterases was measured in a total volume of 300 µL reaction mixture containing oxime and enzyme suspended in 0.1 M phosphate buffer, 0.3 mM DTNB, and ATCh in the 0.1–0.7 mM concentration range. Enzyme activity was tested with a minimum of three oxime concentrations at each substrate concentration. The inhibition constant (*K*_i_) values were evaluated from the experimental data according to the Hunter-Downs equation (Kovarik et al. 2007b). Reversible inhibition experiments were performed at 25 °C, using the Ellman spectrophotometric method at 412 nm on a Tecan Infinite M200PRO microplate reader (Tecan Austria GmbH, Salzburg, Austria), and the results were analyzed with Prism 9 software (GraphPad by Dotmatics, Boston, MA, USA).

### Ex vivo scavenging of cyclosarin

Scavenging of cyclosarin was tested in human whole blood (WB) in the presence of selected oximes. WB was supplemented with 360 nM of purified human plasma BChE and then inhibited with cyclosarin in two ratios, 1:10 and 1:100, corresponding to 3.6 μM and 36 μM cyclosarin which approximate 1 and 10 × LD_50_ dose in mice (*s.c.*). The LD₅₀ of cyclosarin (536.1 µg/kg) was previously determined in our laboratory for a separate project (unpublished data). When 95–100% inhibition of WB was achieved, oxime was added (100 μM). An aliquot was taken at a designated time point for measuring cholinesterase activity with ATCh (1 mM). The same treatment was carried out with WB without exogenous BChE. Control was activity of cholinesterases measured in uninhibited WB, and in WB enriched with BChE-oxime or oxime only. Inhibition of WB by cyclosarin was 100% throughout the experiment to ensure that reactivation was not an artifact, due to, e.g., poor stability of cyclosarin in blood. Measurements were performed at 25 °C and wavelength of 436 nm on a CARY 300 spectrophotometer (Varian Inc., Mulgrave, Australia) using the Ellman method (Ellman et al. 1961), and results were analyzed using the one-phase exponential association model with Prism 9 software (GraphPad, Dotmatics, Boston, MA, USA).

### Molecular modeling

The chain A in the crystal structure of the A*B homodimer of human BChE (PDB ID 2PM8; Ngamelue et al. 2007) was used as a ligand receptor using Clean Protein protocol in Discovery Studio v21.1 (BioVia, San Diego, CA, USA) and adjusted with a CHARMm forcefield including Momany-Rone partial charge method (Brooks et al. 1983; Momany and Rone 1992). The binding site was defined as a 12 Å sphere radius (Maraković et al. 2020, 2021). The docking protocol was set to keep 20 diverse ligand poses in the rigid receptor. The refined pose minimization was the full potential minimization with a final minimization gradient tolerance set to 0 kcal/(mol Å).

The model of cyclosarin-inhibited BChE was prepared by superposition of crystal structure of tabun-inhibited human BChE (PDB ID 3DJY; Carletti et al. 2008), and cyclosarin-inhibited mouse AChE (PDB ID 3ZLU; Artursson et al. 2013) and translation of the cyclosarin moiety from mouse AChE to the Oγ of Ser198 of BChE after removal of tabun conjugate (Maraković et al. 2020).

Molecular modeling of oxime in a near-attack conformation in the cyclosarin-inhibited BChE was performed according to the procedure described previously (Šinko 2023). Cyclosarin-oxime conjugate was docked in BChE Ser198Gly in silico mutant. The pose with the highest overlap of phosphorus-attached substituents with ones in the cyclosarin-inhibited BChE was selected for the generation of the ternary complex between cyclosarin-inhibited BChE and oxime without cyclosarin conjugate. This complex was submitted to full system minimization with a minimization gradient tolerance set to 0.1 kcal/(mol Å).

Molecular dynamics simulation was performed using Standard Dynamics Cascade in Discovery Studio v21.1 (BioVia, San Diego, CA, USA) after structure minimization, 20 steps of Steepest Descent minimization [RMS gradient is 1.0 kcal/(mol Å)] followed by 20 steps of Adopted Basis Newton-Rapson minimization [RMS gradient is 0.1 kcal/(mol·Å)]. Standard Dynamics Cascade starts with a heating stage (*t* = 4 ps) to the target temperature of 310 K. Next, the equilibration stage duration was 20 ps followed by the final production step with a simulation time of 10,000 ps at temperature 310 K, and the save results interval was set to 100 ps. The constraints of the production step included constant pressure and temperature dynamics (NPT). A spherical cut-off was used to treat long-range electrostatics. No implicit solvent model was used during the molecular dynamic simulation.

## Results and discussion

### Oxime library in reactivation of phosphylated BChE

For the initial reactivation screening of human BChE inhibited with cyclosarin, sarin, VX, and tabun, we utilized the oxime library of 62 click-chemistry triazole-annulated oximes (group A), and the building blocks, which were structurally divided into 5 simple pyridinium (group B), 17 complex *N*-alkyl substituted pyridinium (group C), and 27 miscellaneous oximes (group D) (Kovarik et al. 2019b). Oxime potency, estimated in terms of % BChE inhibition and reactivation over 24 h, as well as reactivation rate at a 1 mM oxime concentration and the chemical structures, are provided in Table 1. Among the tested oximes, triazole-annulated compounds exhibited a stronger inhibitory effect on human BChE compared to the other oxime groups. For 20 oximes in Group A, the inhibition ranged from 50 to 86%, indicating a high binding affinity of BChE for these compounds, with estimated dissociation inhibition constants lower than the oxime concentration of 10 µM. This trend is consistent with our previous findings, where both AChE and BChE showed exceptionally high affinity for click-chemistry-derived oximes (Maček Hrvat et al. 2020). Furthermore, the percentages of reactivation observed in 24 h at a 1 mM oxime concentration (shown in Table 1) reveal a substantial number of oximes that successfully reactivated BChE phosphorylated with various NAs. As expected, significant variability in oxime efficacy was observed between NA-BChE conjugates, like previously reported for the same oxime library in the reactivation of phosphorylated human AChE (Kovarik et al. 2019b). Notably, approximately 60 oximes, primarily triazole-annulated and complex pyridinium oximes, successfully reactivated sarin-inhibited BChE, restoring over 80% of its enzyme activity. For cyclosarin-inhibited BChE, 15 triazole-annulated compounds, along with mono-pyridinium oxime 5B, achieved similar results. However, in the case of VX, only three triazole oximes, 19A, 26A, and 1D, reactivated phosphonylated BChE to a significant degree. Upon reviewing Table 1, it is obvious that tabun–BChE conjugate was the most resistant to the reactivation with only five oximes managing to reactivate the conjugate up to 75%.

**Fig. 2:**
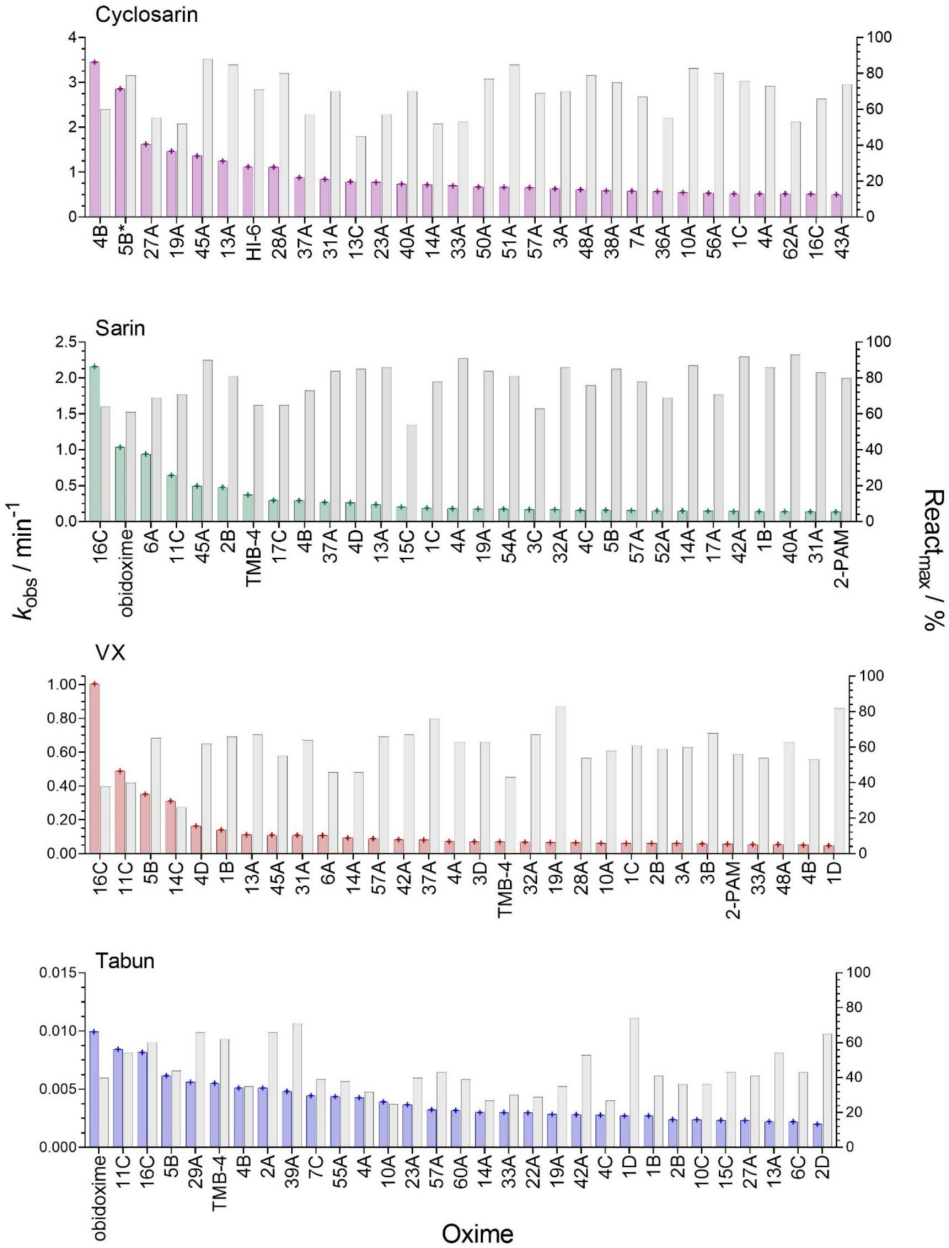
Reactivation screening of NA-inhibited human BChE with top 30 oximes ranked by the observed first-order reactivation rates, *k*_obs_ (left y-axe, colored columns). Maximal reactivation percentage, React_max_ (right y-axe, gray columns) were determined in 24 h. Oxime concentration was 1 mM, except that oxime 5B was 0.1 mM in reactivation of cyclosarin-inhibited BChE (marked with an asterisk) (color figure online)

The top 30 oxime reactivators of four NA–BChE conjugates specified by screening the tested oxime library were sorted by observed reactivation rates (*k*_obs_) and presented in Fig. 2. As it is evident from the y-axes, the most potent reactivators were found for BChE inhibited with cyclosarin, with observed reactivation rates being 1.5, 3.5, and 350 times lower for sarin, VX, and tabun, respectively. This observation confirms that the reactivation efficacy of oximes is directly related to structure of the NA conjugate: *O*-isopropyl methyl, *O*-ethyl methyl, and *O*-cyclohephtyl methyl phosphate (sarin, VX, and cyclosarin, respectively) versus *O*-ethyl phosphoroamidate (tabun) as it was shown previously (Radić et al. 2013; Zandona et al. 2020; Kovarik et al. 2019b, 2004; Horn et al. 2015; Zorbaz et al. 2018a). Furthermore, in the comparison of oxime potencies among the four NAs, there appears to be a direct correlation between the reactivation rates and oxime structures. Generally, mono-pyridinium oximes were faster reactivators of NA-inhibited BChE than triazole oximes. Among the leading reactivators of the cyclosarin–BChE conjugate were two o*rtho*-pyridinium oximes with the hexyl (5B), and pentyl (4B) substituents. In contrast, highest reactivation rates of sarin, VX, and tabun-inhibited BChE were obtained with *meta*-pyridinium oximes having hex-5-ynyl (16C) and pent-4-ynyl substituent (11C).

Triazole-annulated oximes 45A and 13A, both bis-pyridinium bis-oximes, were among the top reactivators of cyclosarin, sarin, and VX–BChE conjugate. In oxime 45A, the oxime group is in *para* position on each of the two pyridinium rings, while in oxime 13A, the oxime group is in *para* position on one ring and in *ortho* position on the other ring. Both oximes have an 8.4 Å distance between their two quaternary nitrogen atoms, equivalent to 8 methylene groups in the extended chain conformation. In our previous study, it was found that approximately 8 Å (or more) between two quaternary nitrogens enables optimal positioning of the reactivator in the tabun-inhibited AChE gorge (Kovarik et al. 2019b). In contrast, the mono-quaternary triazole-annulated oxime, 29A, with a 7 methylene distance, was pointed out as the best reactivator of tabun-inhibited BChE (Fig. 2).

Among the miscellaneous oximes in group D, only mono-imidazolium oximes 4D, 1D, and 3D exhibited the potency to reactivate VX- and sarin-inhibited BChE up to 80%. However, their reactivation rates were slower compared to some of the most efficient reactivators of VX- or sarin-inhibited BChE (Fig. 2; Table 1), or imidazolium oximes previously reported as efficient reactivators of VX-inhibited BChE (Katalinić et al. 2016).

The novel oxime library clearly exhibited significantly higher potential in reactivating cyclosarin, VX, and sarin phosphonylated BChE compared to the standard oximes. For example, the cyclosarin-BChE conjugate was effectively reactivated with two mono-pyridinium oximes (5B, 4B) and two triazole-annulated oximes (45A, 13A) that restored more than 60% activity with a higher reactivation rate than HI-6, the most efficient standard oxime (Fig. 2; Table 1). In the case of sarin-inhibited BChE, pyridinium oxime 16C reactivated the enzyme at a twofold higher rate than the most efficient standard oxime, obidoxime. Moreover, for VX and cyclosarin, obidoxime did not even rank among the top 30 oxime reactivators. In the reactivation of tabun–BChE conjugate obidoxime was similar or more effective in terms of reactivation rate, but its reactivation capacity did not exceed 40%, while for half of the new library oximes, it was > 40%. The FDA-approved oxime 2-PAM was generally not an effective reactivator of NA-BChE conjugates. Yet, it ranked among the top 30 reactivators in the case of sarin- and VX- inhibited BChE, reactivating 80% and 60% of BChE activity, respectively, however, at a much slower rate compared to the most potent reactivators (Fig. 2). This observation is particularly interesting, as the most potent BChE reactivators such as 5B, 4B, 16C, 11C, etc. are structural analogs of 2-PAM (Figs. 1 and 2). Another standard oxime, TMB-4, was able to reactivate sarin- and tabun-inhibited BChE achieving about 60% reactivation, but at a low rate.

### Detailed reactivation kinetics of phosphylated BChE with mono-pyridinium oximes

Based on the initial screening, we selected the most potent reactivators for detailed reactivation kinetics of BChE inhibited with cyclosarin, sarin, VX, and tabun (Fig. 1), and the following parameters were determined: maximum reactivation rate constant (*k*_2_), the apparent dissociation constant (*K*_OX_), overall reactivation rate constant (*k*_r_), maximal percentage of reactivation (React_max_), and time (*t*) in which reactivation maximum was achieved (Table 2).

**Table 2:** Reactivation of phosphylated BChE by selected oximes

NA	Oxime	*k*_2_ (min^−1^)	*K*_OX_ (µM)	*k*_r_ (M^−1^ min^−1^)	React_max_, %	*t* (min)
Cyclosarin	4B	5.37 ± 0.74	300 ± 122	17,890 ± 5177	60	5
	5B	11.67 ± 1.46	342 ± 79	34,120 ± 4144	85	2
	16C	0.63 ± 0.06	139 ± 47	4469 ± 1229	65	15
	Obidoxime	0.57 ± 0.06	348 ± 92	1628 ± 295	65	30
	2-PAM^a^	0.08 ± 0.01	1200 ± 240	65 ± 10	80	60
	HI-6^b^	–	–	780 ± 30	90	30
Sarin	4B	0.30 ± 0.05	770 ± 297	386 ± 84	75	10
	5B	0.18 ± 0.02	71 ± 20	2512 ± 573	85	45
	16C	2.1 ± 0.2	223 ± 33	9439 ± 1317	70	10
	Obidoxime^c^	–	–	1498 ± 73	60	25
	2-PAM	0.12 ± 0.02	1057 ± 350	113 ± 20	85	100
	HI-6^b^	0.04 ± 0.02	270 ± 200	150 ± 40	100	300
VX	2B	0.15 ± 0.04	1411 ± 460	105 ± 21	60	40
	4B	0.05 ± 0.01	149 ± 107	326 ± 191	50	150
	5B	0.90 ± 0.23	1593 ± 610	567 ± 75	65	15
	14C^d^	nd	nd	nd	30	35
	16C^d^	nd	nd	nd	40	15
	Obidoxime^a^	–	–	92 ± 7	70	300
	2-PAM^a^	0.09 ± 0.03	2680 ± 1110	35 ± 5	70	120
	HI-6^e^	0.12 ± 0.03	370 ± 230	330 ± 230	85	120
Tabun	4B	0.0069 ± 0.0009	1037 ± 283	6.7 ± 0.9	35	13 h
	5B^f^	0.0022 ± 0.0001	49 ± 19	45 ± 18	50	10 h
	16C	0.0050 ± 0.0009	791 ± 336	6.4 ± 1.6	60	6 h
	Obidoxime	0.0122 ± 0.0037	4170 ± 1974	30 ± 5	70	6 h
	2-PAM^a^	0.0011 ± 0.0002	1270 ± 530	0.9 ± 0.4	40	12 h
	HI-6^g^	0.0016 ± 0.0004	1,800 ± 900	0.9 ± 0.5	25	

Kinetic parameters (± S.E.): maximal reactivation rate constant (*k*_2_), the apparent dissociation constant (*K*_OX_), and overall reactivation rate constant (*k*_r_), the maximal percentage of reactivation (React_max_), and time (*t*) in which maximal reactivation was achieved were determined from at least three experiments at 25 °C

^a^Zorbaz et al. 2019

^b^Zorbaz et al. 2018a

^c^Linear dependence of *k*_*obs*_ and oxime concentration

^d^Not determined

^e^Katalinić et al. 2016

^f^Kovarik et al. 2019b

^g^Lucić Vrdoljak et al. 2006

Reactivation of cyclosarin–BChE conjugate was very successful with all three selected mono-pyridinium oximes. Among these, oxime 5B achieved an impressive 85% recovery of BChE activity within 2 min, as indicated by a particularly high maximal reactivation rate constant, *k*_2_ (Table 2). The reactivation efficiency, in terms of *k*_r_, was primarily attributed to *k*_2_, while binding affinity (1/*K*_OX_) was generally similar between all tested oximes. The exception was 2-PAM for which *K*_OX_ was about four times higher in comparison to other tested oximes. Ultimately, the selected pyridinium oximes exhibited improved reactivation capacity over 2-PAM due to both increased productivity and lower binding affinity. Additionally, our findings indicate that the reactivation potency, in terms of the overall reactivation rate (*k*_r_), increased with the length of alkyl substituent of *ortho*-oximes (5B > 4B > 2-PAM) (Fig. S1).

The sarin–BChE conjugate was most efficiently reactivated with oxime 16C, which has the oxime group in *meta* position and hex-5-ynyl substituent on the pyridinium ring (Table 2). It is important to mention that the reactivation curves exhibited a biphasic response with an initial burst, where reactivation seemed to accelerate within a narrow concentration range (0.2–0.4 mM), a phenomenon reported previously (Zorbaz et al. 2018b) (Fig. S2). Although we did not further investigate this outcome, the overall reactivation rate constant (*k*_r_) of 16C was highly correlated with the maximal reactivation rate constant (*k*_2_) as in the case of cyclosarin. In contrast, the notable reactivation potency of 5B appears to be a result of its low *K*_OX_, as its *k*_2_ was ten times lower than that of 16C. When compared to standard oximes, 16C and 5B demonstrated a higher potential to reactivate sarin–BChE conjugate. While both obidoxime and HI-6 could be noted as efficient reactivators (due to high *k*_r_ and 100% of reactivation, respectively), the reactivation of sarin-inhibited BChE was up to 60% by obidoxime, and 50-fold slower with HI-6 in comparison to oxime 16C. It is worth mentioning that, both oximes, 5B and 16C exhibited higher reactivation rates than quinuclidinium or pyridinium oximes reported previously (Zandona et al. 2020; Zorbaz et al. 2019).

Among the selected oximes, 5B was the most efficient reactivator of VX-inhibited BChE, restoring up to 65% of the BChE activity in 15 min (Table 2). Its productive binding, indicated by *k*_2_ value, was 6- to 18-fold higher than for oximes 2B or 4B, respectively. However, due to the apparent dissociation constant in the mM range, its reactivation potency was not proportionally related to *k*_2_. Reactivation of the VX–BChE conjugate was also analyzed with complex *N*-alkyl substituted pyridinium oximes 16C and 14C that were identified as promising reactivators in screening (Fig. 2). Unfortunately, both oximes displayed limited efficacy in reactivation reaching a reactivation maximum of 40%. Moreover, at concentrations below 1 mM, the reactivation efficacy dropped to just 20%. Thus, while 5B appears to be better reactivator than standard oximes, its efficacy is about ten times lower than that of benzimidazole oximes, which have been reported as efficient reactivators of the VX–BChE conjugate (Katalinić et al. 2016).

Tabun–BChE conjugate was quite resistant to reactivation by pyridinium oximes (Table 2). A maximum of 60% reactivation was observed with oxime 16C after several hours. Generally, reactivation rates of tabun-inhibited BChE were slow likely due to steric hindrance from the *N,N*-dimethyl substituent. However, along with the steric hindrance and unproductive reactivation indicated by low *k*_2_ values, high dissociation constants implied that binding affinity at the BChE catalytic serine was also affected in the case of tabun moiety. A comparison of *k*_r_ values listed in Table 2 indicates that oxime 5B was the most effective due to low *K*_OX._ In contrast, obidoxime, while having the highest potency for the nucleophilic displacement of the phosphorus moiety (*k*_2_), reactivated tabun–BChE conjugate up to 70% despite poor binding affinity. However, selected oximes did not improve the effectiveness of pyridinium oximes reported previously (Zorbaz et al. 2019; Katalinić et al. 2018). It is interesting to mention previous reactivation results on tabun-inhibited AChE mutant, Y337A, since this mutation defines a residue found in BChE at the presumed position of the choline-binding site. About 90% of mutant Y337A–tabun conjugate activity was restored by oxime 5B within 20 min, primarily due to 100-fold higher *k*_2_ than herein obtained for BChE (Kovarik et al. 2019a). Although such improvement in reactivation with 5B seen for the mutant was not observed for BChE, it is important to note that 5B is one of the most efficient reactivators of tabun–BChE conjugate reported so far (Katalinić et al. 2016, 2018, 2017; Lucić Vrdoljak et al. 2006; Zorbaz et al. 2019, 2018a; Horn et al. 2015; Čalić et al. 2008).

### Triazole-annulated bis-pyridinium bis-oximes and mono-pyridinium mono-oximes as reactivators of cholinesterases inhibited with cyclosarin

In our previous study with the click-chemistry oxime library, we identified three 1,4-triazole bipyridinium oximes 3A, 14A, and 22A, as efficient reactivators of the cyclosarin-inhibited AChE (Kovarik et al. 2019b). To explore the potential of dual-use reactivators that could effectively reactivate both enzymes, we tested the reactivation of AChE with the best reactivators of BChE (4B, 5B, 16C), and reactivation of BChE with 3A, 14A, and 22A oximes in case of inhibition by cyclosarin. Reactivation kinetics parameters given in Table 3 clearly showed that high recovery of AChE activity ranging from 70 to 100% was achieved with all selected oximes. However, 1,4-triazole bipyridinium oximes exhibited maximal reactivation rates up to 90 times higher than those of mono-pyridinium oximes (Table 3; Fig. S3). When combined with a low *K*_OX_, as seen with 1,4-triazole bipyridinium oxime 14A, the overall reactivation rate was comparable to HI-6, the most potent reactivator of cyclosarin-inhibited AChE (Zorbaz et al. 2018a). This reactivation capacity aligns with the findings of our previous study (Kovarik et al. 2019b).

**Table 3:** Reactivation of cyclosarin-inhibited cholinesterases by selected oximes

Enzyme	Oxime	*k*_2_(min^−1^)	*K*_OX_ (µM)	*k*_r_ (M^−1^ min^−1^)	React_max_ (%)	*t* (min)	*K*_i_ (µM)
AChE	3A	0.20 ± 0.07	61 ± 45	3234 ± 1335	100	15	1.92 ± 0.13
	14A	0.73 ± 0.1	36 ± 14	20,220 ± 5337	100	10	7.91 ± 0.42
	22A	0.59 ± 0.15	188 ± 72	3132 ± 379	100	10	2.02 ± 0.12
	4B	0.008 ± 0.001	169 ± 86	49 ± 18	95	360	10.92 ± 1.27
	5B	0.009 ± 0.001	89 ± 50	98 ± 46	100	300	16.92 ± 1.40
	16C	0.018 ± 0.002	46 ± 29	404 ± 230	70	180	11.17 ± 1.25
	2-PAM^a^	0.05 ± 0.002	1178 ± 138	43 ± 3	100	120	210 ± 45^b^
	HI-6^bc^	–	–	22,100 ± 1300	100	1	46 ± 4.3 ^b^
BChE	3A	1.15 ± 0.32	1356 ± 570	850 ± 130	80	20	10.36 ± 0.79
	14A^ca^	–	–	725 ± 38	70	30	38.62 ± 2.20
	22A	0.90 ± 0.16	1268 ± 336	713 ± 73	75	20	6.62 ± 1.29
	4B	5.37 ± 0.74	300 ± 122	17,890 ± 5177	60	5	23.48 ± 4.37
	5B	11.67 ± 1.46	342 ± 79	34,120 ± 4144	80	2	26.75 ± 3.23
	16C	0.63 ± 0.06	139 ± 47	4469 ± 1229	65	15	37.92 ± 6.89
	2-PAM^d^	0.08 ± 0.01	1200 ± 240	65 ± 10	80	60	140 ± 16 ^b^
	HI-6^bc^	–	–	780 ± 30	90	30	420 ± 100 ^b^

Kinetic parameters (± S.E.): maximal reactivation rate constant (*k*_2_), the apparent dissociation constant (*K*_OX_), and overall reactivation rate constant (*k*_r_), the maximal percentage of reactivation (React_max_), and time (t) in which maximal reactivation was achieved. Reversible inhibition constants (*K*_i_) for AChE and BChE with selected oximes were also determined. These parameters were determined from at least three experiments at 25 °C

^a^Zorbaz et al. 2018b

^b^Zorbaz et al. 2018a

^c^Linear dependence of *k*_*obs*_ vs. oxime concentration in the studied concentration range

^d^Zorbaz et al. 2019

In the case of the cyclosarin–BChE conjugate, selected 1,4-triazole oximes recovered up to 80% of BChE activity which corresponds to the initial screening analyses (Fig. 2). However, despite the high reactivation maximum, due to high *K*_OX_, the overall reactivation rates of oximes 3A, 14A, and 22A were significantly lower than with mono-pyridinium oximes 4B and 5B (Table 3). Generally, the 1,4-triazole bipyridinium oximes were superior in the reactivation of AChE, while mono-pyridinium mono-oximes were superior in reactivation of BChE, when inhibited with cyclosarin. This is emphasizing reactivation selectivity governed by the difference of active sites shown in the previous studies (Kovarik et al. 2015, 2019a, 2004; Maček Hrvat et al. 2020; Katalinić et al. 2018). It is worth mentioning that oximes 14A, 3A, and 22A showed a higher reactivation potency for both AChE and BChE inhibited with cyclosarin compared to 2-PAM, and exhibited a similar capacity to HI-6 for inhibited BChE.

To investigate the binding affinity of native AChE and BChE for the selected oximes and how it is affected by the conjugation of phosphorus moiety, we determined reversible inhibition constants (*K*_i_) presented in Table 3. The selected oximes inhibited both enzymes at a lower micromolar range, with AChE showing up to a fivefold preference for binding. Triazole bis-pyridinium bis-oximes exhibited slightly better potency as inhibitors of native cholinesterases compared to mono-pyridinium mono-oximes. This difference may be attributed to the reduced number of π–π and other non-covalent interactions between mono-pyridinium oximes and the active gorge of cholinesterases. Nevertheless, both classes of oximes were better reversible inhibitors compared to standard oximes, HI-6, and 2-PAM. This finding agrees with the previous studies that showed that triazoles as well as bulkier oximes can synergistically contribute to the overall binding energy (Manetsch et al. 2004; Maček Hrvat et al. 2020). Namely, the phenyltetrahydroisoquinoline triazole aldoximes inhibited AChE 1000 times more effectively than selected triazole bis-pyridinium bis-oximes likely due to the larger number of enzyme–oxime interactions (Maček Hrvat et al. 2020).

As expected, the phosphonylated enzymes had a significantly lower binding affinity for the six selected oximes than native enzymes. The affinity (1/*K*_OX_) of the phosphonylated BChE for triazole-annulated oximes, 3A, and 22A, decreased up to 200 times compared to the affinity (1/*K*_i_) of native BChE (Table 3). This decrease in binding affinity is probably due to the conformational changes in the active site, and steric hindrance by the OP moiety at the catalytic serine affecting oxime’ embedding and orientation, as suggested in the previous studies (Gorecki et al. 2024; Katalinić et al. 2018; Bennion et al. 2015; Čadež et al. 2021). This analysis highlights that for successful reactivation, reactivating oxime antidotes can rely on moderate binding affinity and a high dephosphorylation rate (Kovarik et al. 2004; Worek et al. 2016; Lindgren et al. 2022).

### Bioscavenging of cyclosarin

**Fig. 3:**
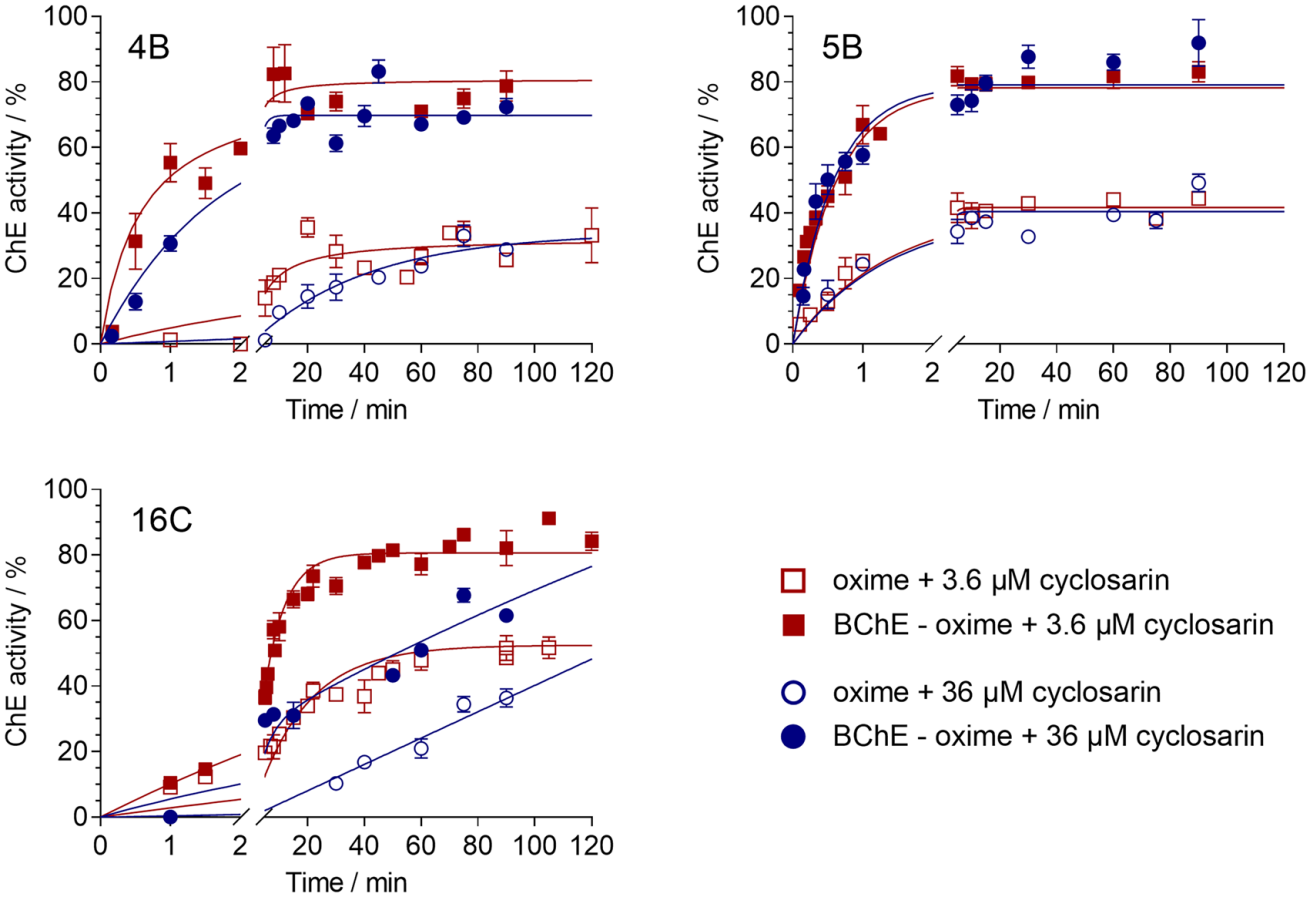
Ex vivo detoxification of cyclosarin (3.6 μM, red curve, and 36 μM, blue curve) in whole blood supplemented with BChE (360 nM) and oximes 4B, 5B, and 16C (100 µM) shown as activity with oxime (open symbols), and with the addition of BChE and oxime (full symbols). The mean of at least six experiments ± SEM is presented. Curves are generated using the one-phase exponential association model (color figure online)

**Fig. 4:**
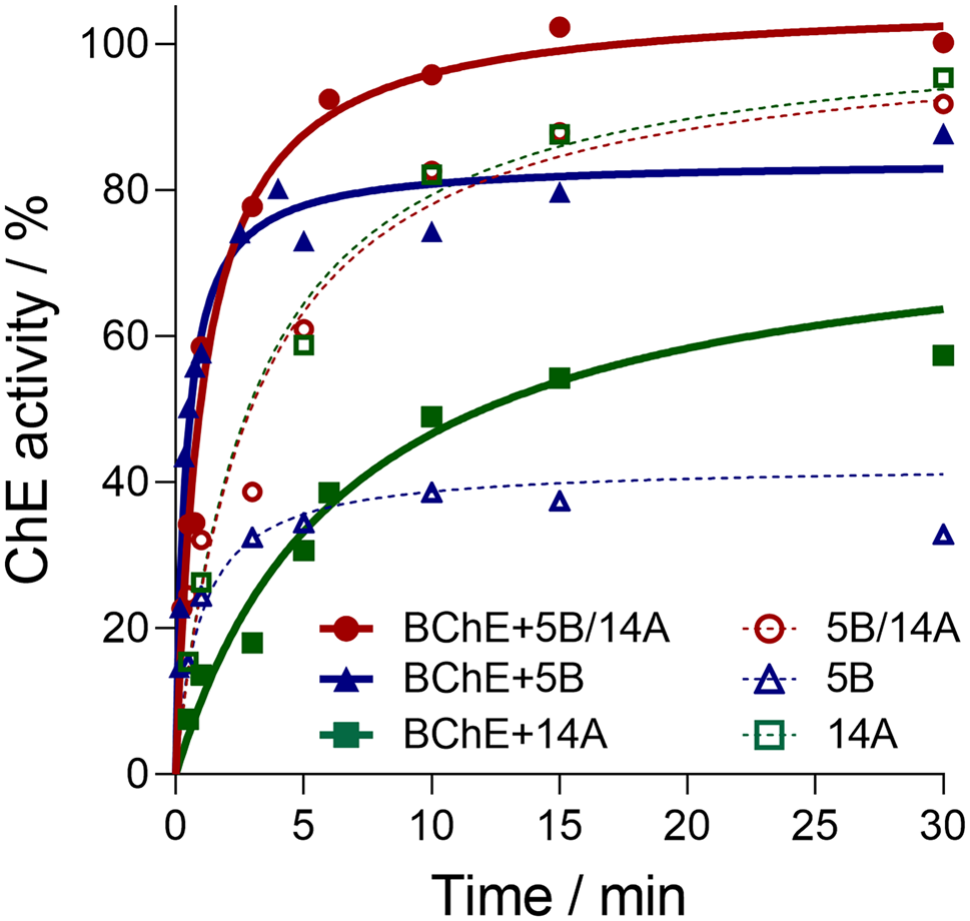
Ex vivo detoxification of cyclosarin (36 µM) in whole blood supplemented with BChE (360 nM) and oximes 5B and 14A (100 µM) shown as activity with oxime (dashed curve), and with the addition of BChE and oxime (solid curve). The mean of at least three experiments ± SEM is presented. Curves are generated using the one-phase exponential association model

It is expected that detoxification of NA compounds can be established in whole blood by exogenous cholinesterase-oxime bioscavenger, as demonstrated in the previous studies involving AChE mutants (Kovarik et al. 2015, 2019a; Maček Hrvat et al. 2016) and BChE (Radić et al. 2013; Zandona et al. 2020) with efficient reactivator of either enzyme. Based on the successful reactivation of both BChE and AChE inhibited by cyclosarin we tested bioscavenging of cyclosarin in ex vivo conditions, where human whole blood (WB) was supplemented with BChE and selected reactivators. The increase in total cholinesterase activity (activity of exogenous BChE, erythrocyte AChE, and plasma BChE) proved the degradation of cyclosarin as shown in Figs. 3 and 4.

The most successful degradation of 10- to 100-fold excess of cyclosarin concentration over exogenous BChE was evident with the BChE-5B pair, as expected based on in vitro results (Fig. 3, cf. Table 2). Approximately 70% of the cholinesterase activity was recovered within 2 min, regardless of tested cyclosarin concentrations. Nevertheless, none of the enzyme–oxime pairs achieved 100% recovery of cholinesterase activity in the WB, which was somewhat expected due to the limited capacities of these oximes to reactivate the cyclosarin-inhibited AChE (cf. Table 3).

Therefore, to enable ex vivo reactivation of erythrocyte AChE inhibited with cyclosarin, we combined oxime 14A, as a potent reactivator of the cyclosarin-inhibited AChE, with oxime 5B and monitored the return of cholinesterase activity in WB supplemented with exogenous BChE. As shown in Fig. 4**,** the cyclosarin degradation was completed in less than 15 min after the addition of both 5B and 14A oxime with a 100% recovery of total cholinesterase activity in the case of 100-fold excess of cyclosarin (36 μM concentration) to supplemented exogenous BChE. The recovery of total ChE activity with only oxime 5B or 14A was 80% and 60%, respectively, consistent with the excess of BChE over AChE in this system and the reactivation selectivity (cf. Table 3).

When the WB was not supplemented with BChE, the combination of oximes 5B and 14A enabled recovery up to 85% of cholinesterase activity, but over six times longer period when compared to the WB with exogenous BChE. Furthermore, the maximal recovery of cholinesterase activity with oxime 14A alone was similar to the recovery observed with the combination of two oximes. This could be attributed to the higher concentration of AChE compared to BChE in WB (Reiner et al. 2004; Worek et al. 1999), and the higher affinity of cyclosarin-inhibited AChE for oxime 14A (cf. Table 3).

Similar results were obtained in the presence of 3.6 µM cyclosarin in WB, both with and without BChE supplementation (not shown).

### Molecular modeling of oxime in the cyclosarin-inhibited BChE

**Fig. 5:**
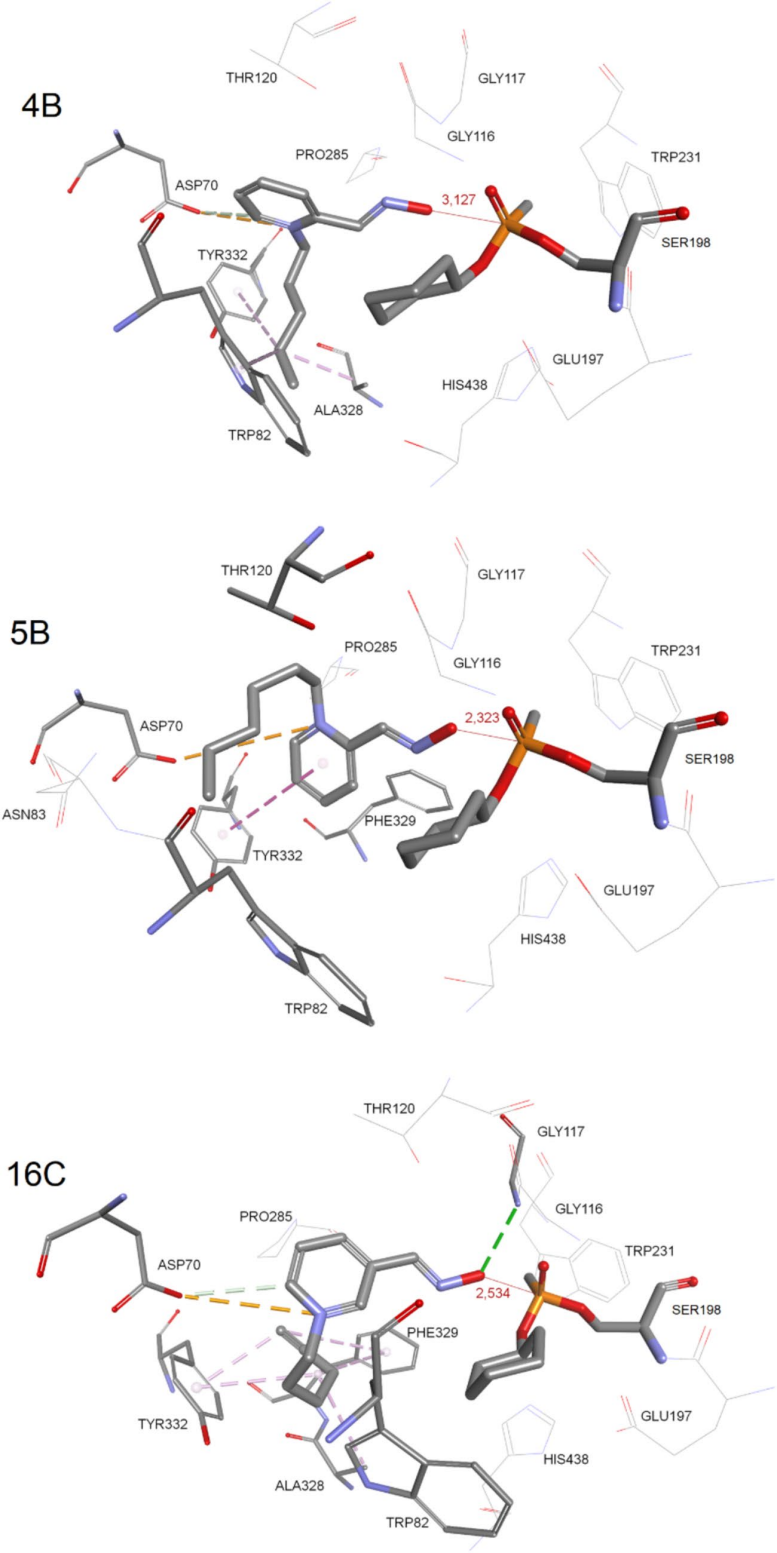
Model of BChE–cyclosarin conjugate (PDB ID 2PM8; Ngamelue et al. 2007) in reversible complex with selected oximes. Interactions of oximes with amino acid residues are represented as dashed lines: hydrophobic (purple), hydrogen bonds (green), and electrostatic (orange) (color figure online)

Modeling of the near-attack conformation of oximes in the cyclosarin-inhibited BChE resulted in the position of the deprotonated atom of the oxime group in line with P and Oγ atoms from catalytic Ser198 (Fig. 5). According to S_N_2 mechanism of reactivation, this orientation of atoms produces a transition state (Maraković et al. 2020). The distance from the deprotonated O atom of the oxime group to the phosphorus atom of cyclosarin conjugate was 3.13 Å, 2.32 Å, and 2.53 Å, for 4B, 5B, and 16C, respectively. It is important to note that their pyridinium rings are in different positions in the native BChE (Fig. S4). In the case of native BChE, the pyridinium ring interacts with Trp82, while in conjugated BChE, this interaction cannot be realized due to the presence of the cyclohexyl ring. As shown in Fig. 5, interactions of 4B included electrostatic interaction and hydrogen bond between pyridinium ring and Asp70, and hydrophobic interaction for stabilization of aliphatic tail with Trp82, Ala328, and Tyr332. Similarly, the pyridinium ring of 5B created electrostatic interaction with Asp70 and hydrophobic interaction with the aromatic ring of Tyr332, while its alkyl substituent was directed toward the backbone of Trp82. Both oximes 16C and 4B formed an electrostatic interaction between the pyridinium ring and carboxyl group of Asp70 accompanied with a hydrogen bond. The unsaturated aliphatic tail of oxime 16C adopts a bent conformation creating a net of hydrophobic interactions with Ala328, Phe329, and Tyr332.

Furthermore, the model of the complex between oxime 5B and the cyclosarin-inhibited BChE was submitted to full system molecular dynamics (*t* = 10 ns) to test the mobility of the near-attack conformation of oxime 5B as well as to overcome restrictions of the applied docking method. Namely, using molecular dynamics, we analyzed the distance between the oxygen of the oxime group and the phosphorus atom of the phosphoester conjugate, which should be up to 4 Å for the transition state and productive reactivation of BChE to occur. Along with this distance, the molecular dynamics trajectory was done for the distance between carbon oxime atoms and Trp82, Tyr332, and Asn83 (Fig. S5). Generally, the change of these distances indicated the stability of interactions and movement of the oxime molecule within the active site. Distance from 5B to Trp82 was stable for about 2/3 of the simulation time (~ 9.2 Å) with a later increase up to ~ 11.5 Å. A similar effect was seen for the distance between Tyr332 and 5B (from 4–5 Å to 6.5 Å). The highest relative fluctuation was observed for Asn83 for which the distance was increased from 3.8 Å to 6.5 Å; an average distance was 5.46 Å. Overall, molecular dynamics showed changes in the 5B position in the active site, but the key distance from the oxygen of the oxime group to the phosphorus atom of the phosphoester conjugate remained stable during simulation time.

It is important to emphasize that oxime orientation within the active site of near attack in the cyclosarin-inhibited BChE revealed different poses from the conformation of oximes in the native BChE due to the repositioning of pyridinium ring (Figs. S1 and S4) causing the elimination of previously obtained interaction between pyridinium ring of oximes and Trp82 (Fig. S4). It is clear how steric effects of cyclohexyl conjugated at the Ser198 modify the binding of oximes due to the reduction of active site volume and restricting interactions of the pyridinium ring with Trp82 from the choline-binding site. Also, pyridinium ring electrostatic interaction and hydrogen bond with Asp70 are not present in the native BChE.

## Conclusion

The oxime library of simple pyridinium and imidazole oximes, and triazole-annulated oximes prepared by the click-chemistry reaction provided a new source of efficient reactivators of BChE inhibited with cyclosarin, sarin, VX, and tabun. Structural analogs of 2-PAM, mono-pyridinium compounds with the hexyl/pentyl alkyl substituent, showed a universal capability to efficiently reactivate cyclosarin-, sarin-, and VX-inhibited BChE. Moreover, these novel oximes reactivate NA–BChE conjugates more effectively than standard oximes.

The cyclosarin-phosphylated BChE was the most prone to reactivation with these oximes. Modeling the near-attack conformation of oxime 5B within the active site of inhibited BChE revealed a different binding pose compared to the non-inhibited BChE, which was attributed to the steric hindrance caused by the cyclosarin moiety. Molecular dynamics simulation resulted in a stable position of its oxime group at the van der Waals distance from the phosphorus indicating a proper position for the nucleophilic attack enabling productive reactivation.

Ex vivo  experiments with the BChE-5B pair showed remarkable recovery of cholinesterase activity, even in the presence of a 100-fold excess of cyclosarin in whole blood. Moreover, exogenous BChE with the simultaneous addition of oximes 5B and 14A, potent reactivators of cyclosarin-inhibited BChE and AChE, respectively, enabled even faster recovery of total cholinesterase activity proving that cyclosarin was degraded in the blood through a continuous cycle of cholinesterases inhibition and efficient oxime-assisted reactivation.

Efficient reactivation demonstrated under both in vitro and ex vivo conditions suggests the potential for further testing of the click chemistry-derived oxime library, and paving the way for the development of pseudo-catalytic bioscavengers based on efficient reactivators of BChE as a promising therapy for NAs poisoning.

## Supplementary Information

Below is the link to the electronic supplementary material.

Supplementary file1 (PDF 735 kb)

## Data Availability

The raw data supporting the conclusions of this article will be made available by the authors upon request.
