# Supplementary material for: Click-chemistry-derived oxime library reveals efficient reactivators of nerve agent-inhibited butyrylcholinesterase suitable for pseudo-catalytic bioscavenging
Source: Arch Toxicol. 2025 Mar 3;99(5):2107–31. doi: 10.1007/s00204-025-03985-6 (PMC12085358; doi:10.1007/s00204-025-03985-6)
Supplement: Supplementary file 1 — Supplementary file1 (PDF 735 kb) [file 204_2025_3985_MOESM1_ESM.pdf]

https://doi.org/10.1007/s00204-025-03985-6

Supplementary material

Click-chemistry-derived oxime library reveals efficient reactivators of nerve agent-inhibited butyrylcholinesterase suitable for pseudocatalytic bioscavenging

Tena Čadež[1], Nikolina Maček Hrvat[1], Goran Šinko[1], Jarosław Kalisiak[2], Zoran Radić[3], Valery V. Fokin[2,4], Karl Barry Sharpless[2], Palmer Taylor[3], and Zrinka Kovarik[1,5,*]

[1] Institute for Medical Research and Occupational Health, Ksaverska cesta 2, Zagreb, Croatia

[2] Skaggs Institute for Chemical Biology, The Scripps Research Institute La Jolla, CA, USA

[3] Skaggs School of Pharmacy and Pharmaceutical Sciences University of California at San Diego La Jolla, CA, USA

[4] Present address: The Bridge@USC and Loker Hydrocarbon Research Institute, University of Southern California, Los Angeles, CA, USA

[5] Faculty of Science, University of Zagreb, Zagreb, Croatia

Contents

S1. Reactivation efficacy of cyclosarin inhibited BChE *in vitro* 2
S2. Biphasic response of cyclosarin inhibited BChE reactivated with oxime 16C 2
S3. Reactivation efficacy of cyclosarin inhibited AChE *in vitro* 3
S4. Molecular modeling 4
S5. Molecular dynamics 5

https://doi.org/10.1007/s00204-025-03985-6

S1. Reactivation efficacy of cyclosarin inhibited BChE *in vitro*

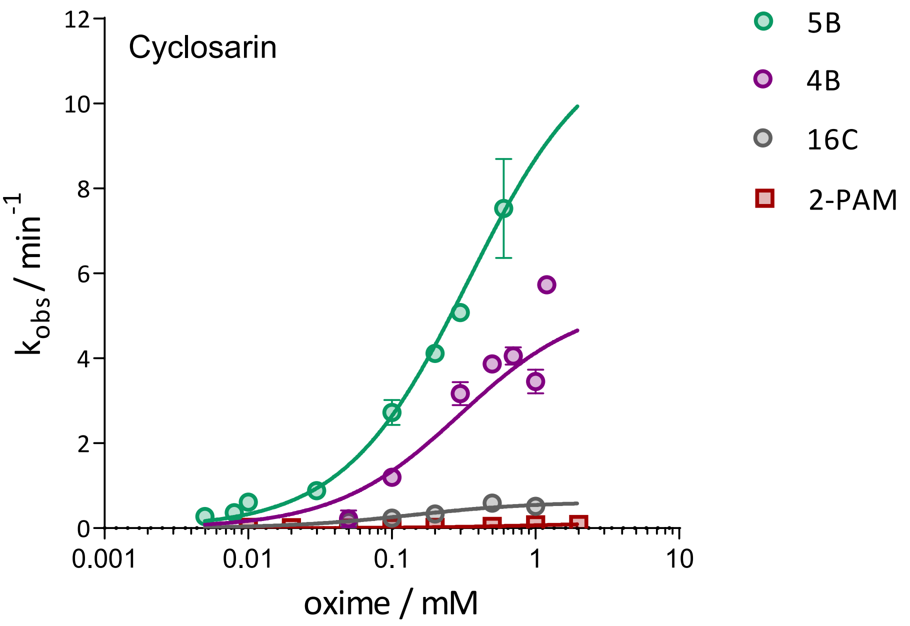

Figure S1. Reactivation kinetics of cyclosarin-inhibited BChE by the selected oximes. The results are expressed as mean ± SEM calculated from at least three experiments.

S2. Biphasic response of cyclosarin inhibited BChE reactivated with oxime 16C

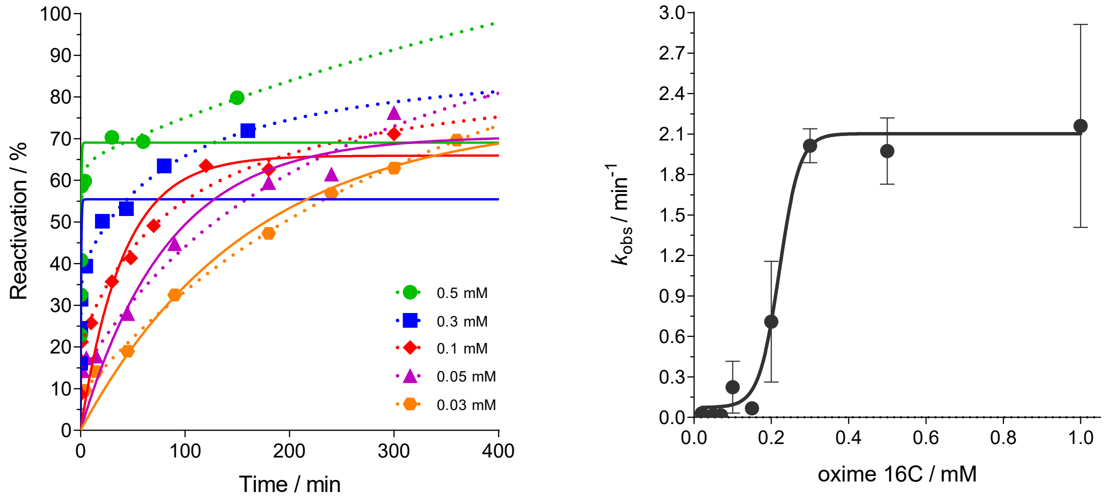

Figure S2. Deviation from the typical hyperbolic model in the reactivation of sarin-inhibited BChE with oxime 16C. The left graph shows changes in reactivation percentage over time, with dependence described by a theoretical single-phase curve (solid line) and a modified two-phase curve (dashed line) for each oxime concentration. The right graph illustrates the dependence of observed reactivation rates on oxime concentration. The mean of at least seven experiments ± SEM is presented.

S3. Reactivation efficacy of cyclosarin inhibited AChE *in vitro*

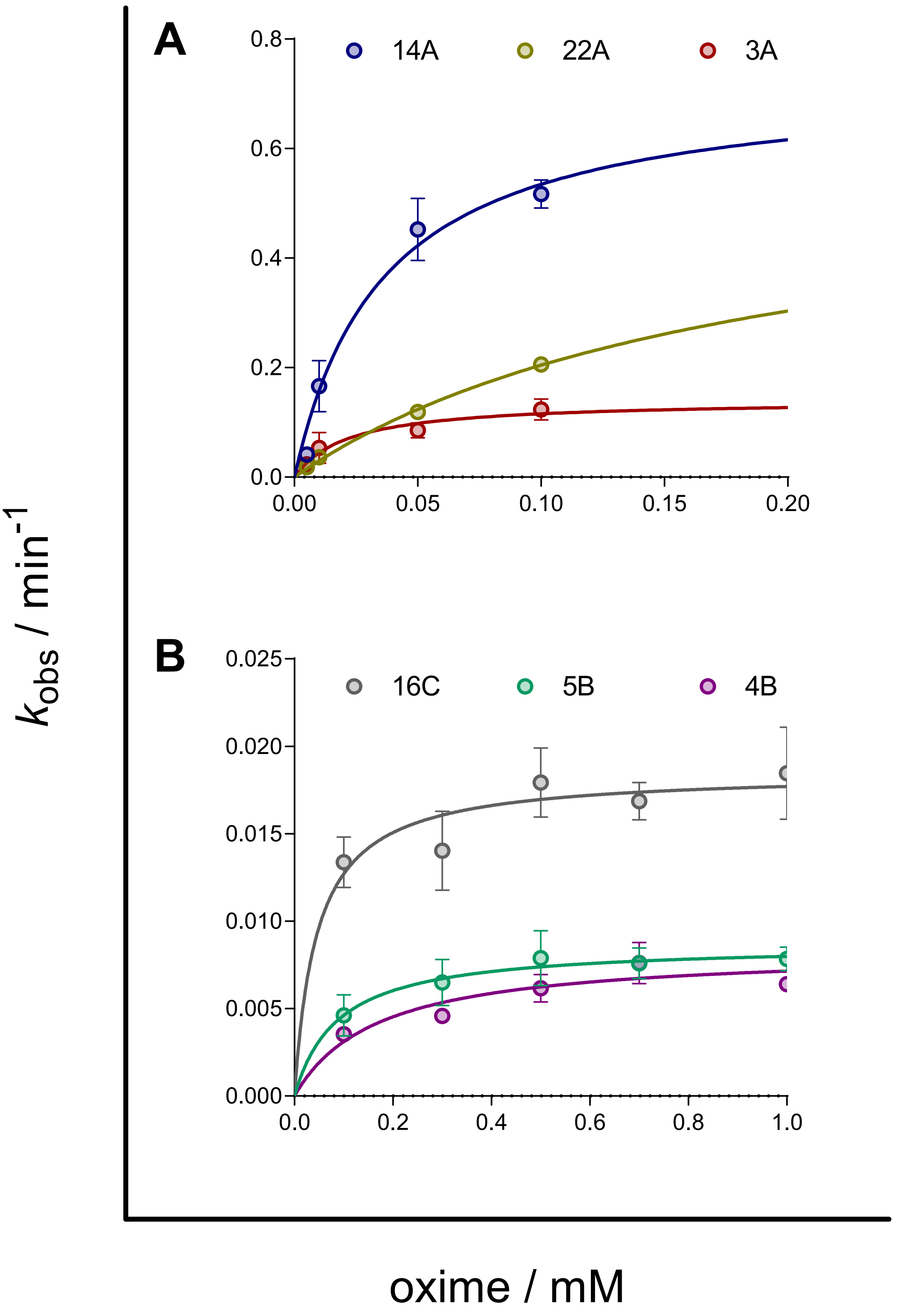

Figure S3. Reactivation kinetics (k_{obs}) of cyclosarin-inhibited AChE determined with (A) bis-pyridinium (14A, 22A or 3A) and (B) mono-pyridinium (16C, 5B or 4B) oximes.

https://doi.org/10.1007/s00204-025-03985-6

S4. Molecular modeling

OXIME

4B

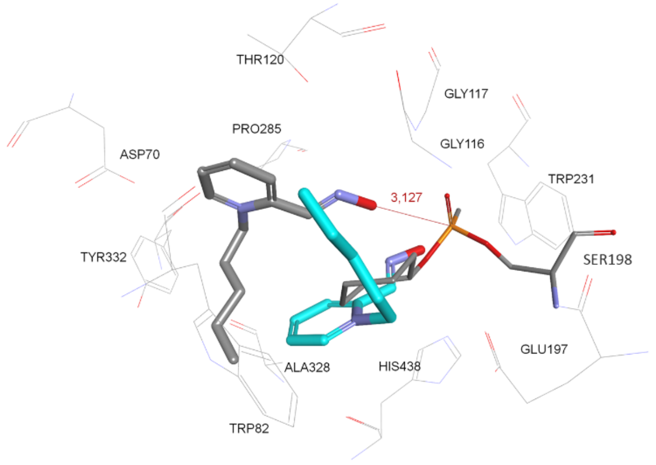

5B

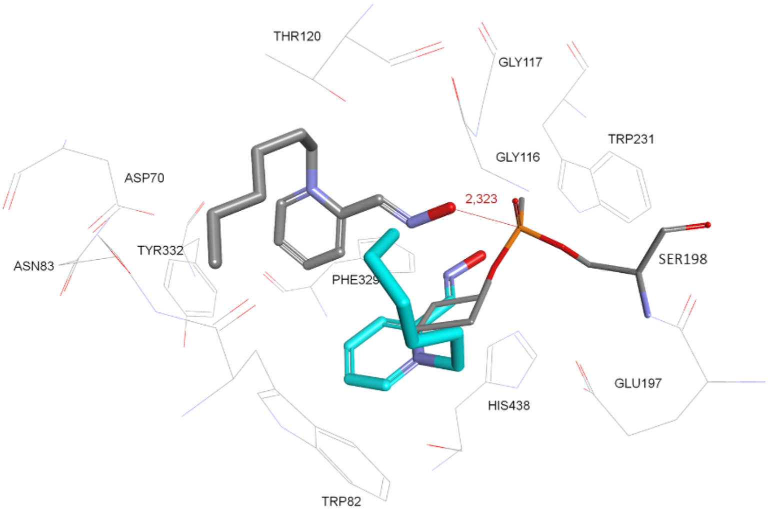

16C

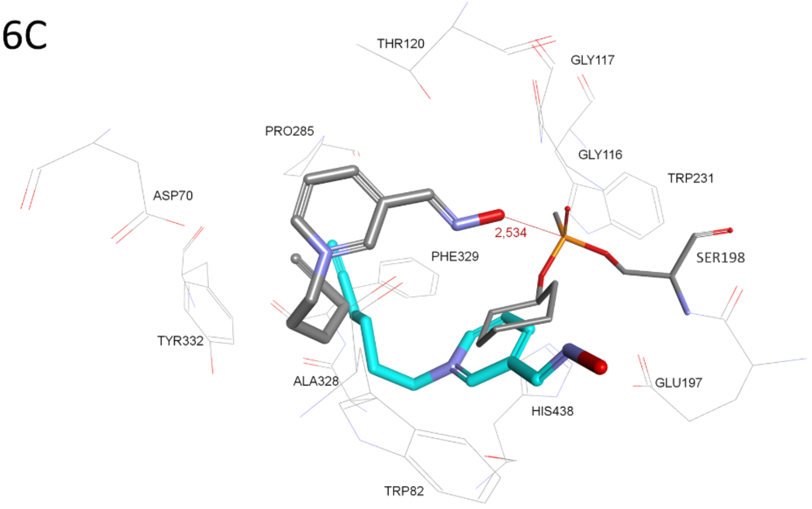

Figure S4. Superposition of binding poses of oximes in the active site of native BChE (blue) and in the near-attack conformation in BChE-cyclosarin conjugate (grey; given in **Figure 5**). The red line indicates the distance of the oxime group from the phosphorus atom of cyclosarin conjugate.

https://doi.org/10.1007/s00204-025-03985-6

S5. Molecular dynamics

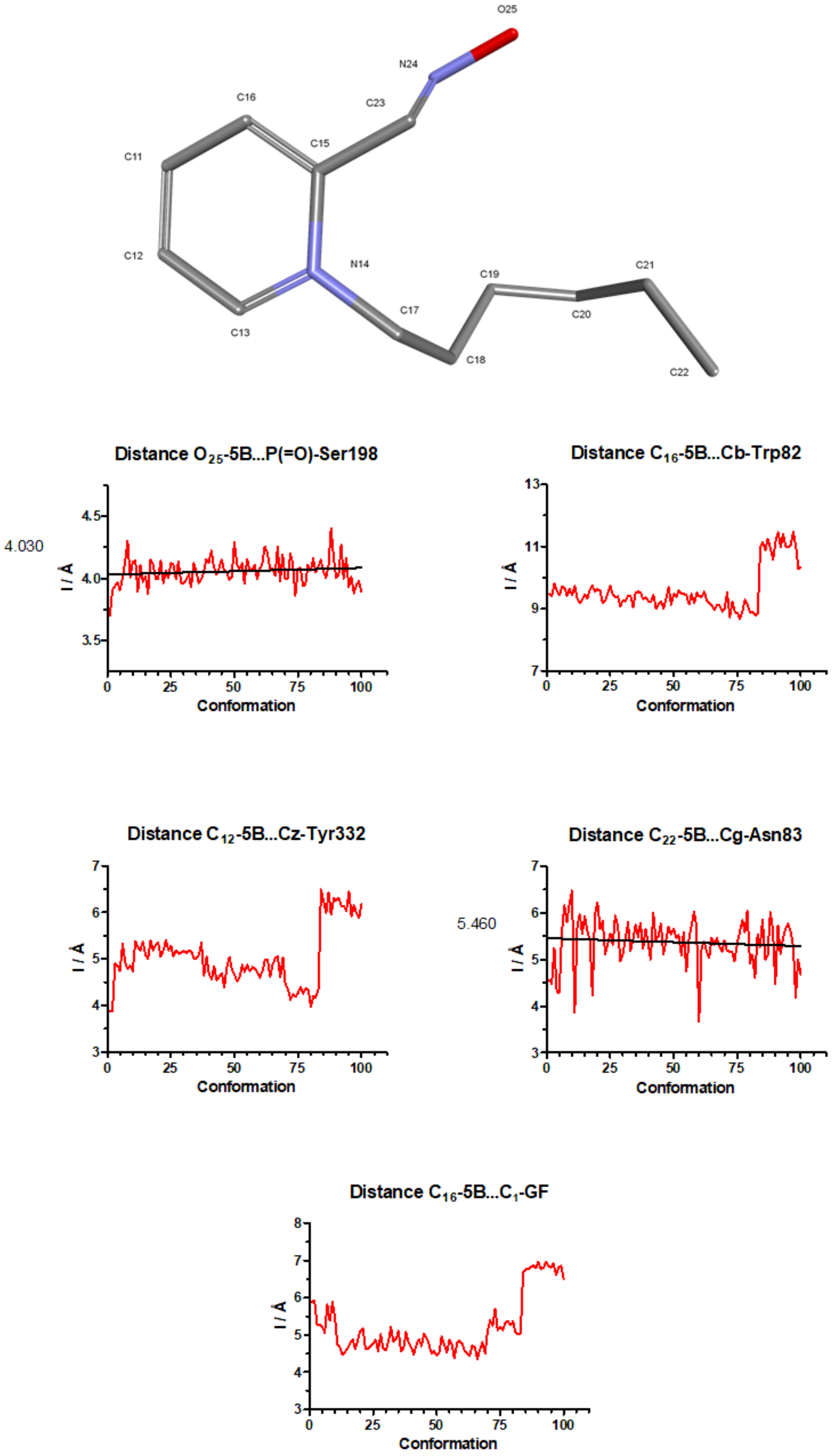

Figure S5. Molecular dynamics simulation (t=10 ns) of complex between an oxime 5B and cyclosarin-conjugate of BChE. Distances between atoms of 5B and selected atoms of BChE active site residues are shown. Distance average value (Å) is listed left of Y-axes.
